# *Candida* species in community-acquired pneumonia in patients with chronic aspiration

**DOI:** 10.1186/s41479-021-00090-x

**Published:** 2021-07-05

**Authors:** Benjamin J. Moss, Daniel M. Musher

**Affiliations:** 1 Baylor College of Medicine, Houston, TX USA; 2 Michael E. DeBakey Veterans Affairs Medical Center, Infectious Disease Section, Room 4B-370, VA Medical Center, Houston, TX 77030 USA

**Keywords:** Pneumonia, *Candida*, Fungal pneumonia

## Abstract

**Background:**

When *Candida* species is found in a sputum culture, clinicians generally dismiss it as a contaminant. We sought to identify cases of community-acquired pneumonia (CAP) in which *Candida* might play a contributory etiologic role.

**Methods:**

In a convenience sample of patients hospitalized for CAP, we screened for “high-quality sputum” by Gram stain (> 20 WBC/epithelial cell) and performed quantitative sputum cultures. Criteria for a potential etiologic role for *Candida* included the observation of large numbers of yeast forms on Gram stain, intracellular organisms and > 10^6^ CFU/ml *Candida* in sputum. We gathered clinical information on cases that met these criteria for possible *Candida* infection.

**Results:**

Sputum from 6 of 154 consecutive CAP patients had large numbers of extra- and intracellular yeast forms on Gram stain, with > 10^6^ CFU/ml *Candida albicans*, *glabrata*, or *tropicalis* on quantitative culture. In all 6 patients, the clinical diagnoses at admission included chronic aspiration. Greater than 10^5^ CFU/ml of a recognized bacterial pathogen (*Streptococcus pneumoniae*, *Staphylococcus aureus*, or *Pseudomonas)* or > 10^6^ CFU/ml of other ‘normal respiratory flora’ (*Lactobacillus* species) were present together with *Candida* spp. in every case. Blood cultures yielded *Candida* in 2 cases, and 1,3-beta-D glucan was > 500 ng/mL in 3 of 3 cases in which it was assayed. Since all patients were treated with anti-bacterial and anti-fungal drugs, no inference about etiology can be derived from therapeutic response.

**Conclusions:**

*Candida* spp. together with a recognized bacterial pathogen or normal respiratory flora may contribute to the cause of CAP in patients who chronically aspirate.

## Background

A standard teaching amongst infectious disease specialists is that pneumonia due to *Candida* species is a “very rare event” [1]. These organisms regularly colonize the mouth, and their numbers can increase if antibiotics are given. When yeast forms are seen on Gram stain and *Candida* are grown in sputum cultures, they are generally dismissed as oropharyngeal contaminants from colonizing organisms. However, in severely immunocompromised patients, *Candida* spp. is known to invade locally causing thrush or, occasionally, pneumonia [1–5].

Definitive diagnosis of *Candida* pneumonia requires visualization of invasive *Candida* forms in the lung parenchyma. Autopsy series from large groups of immunosuppressed patients have documented the infrequent occurrence of *Candida* pneumonia [3–5]. Recent studies, however, have given more attention to a possible pathogenic role in pneumonia for *Candida* in combination with bacterial pathogens [6–9].

In the course of a prospective observational study that documented an important role for normal respiratory flora as the cause of community acquired pneumonia (CAP) [10], we observed a surprising number of patients in whom *Candida* appeared to be playing a contributory role. In each case, chronic aspiration had been identified as a possible cause in the admitting history. In the present paper, we describe the clinical and microbiologic features of these and subsequent cases and propose that *Candida*, together with recognized bacterial pathogens or so-called normal respiratory flora, contributes to CAP more commonly than is generally believed, particularly in patients with a history of chronic aspiration.

## Methods

### Study design

Cases were identified during a prospective study of a convenience sample of patients admitted to the Michael E. DeBakey VA Medical Center between September 1, 2017 and January 31, 2020. On days selected for study, we examined Gram stains of all sputum samples that had been submitted to the clinical microbiology laboratory in the preceding 24 h without first reviewing the medical record. For every sputum categorized as high-quality (> 20 white blood cells (WBC) per epithelial cell, a more rigorous standard than is usually used [11]), we reviewed the medical record to identify patients who had been admitted from the community with a diagnosis of CAP and had submitted the sputum sample within 16 h of antibiotic initiation. The diagnosis of CAP was verified based on the presence of a newly recognized pulmonary infiltrate and at least two of the following findings: subjective or objective fever; increased cough, sputum production or shortness of breath; pleuritic chest pain, rales, or confusion. Patients who had received antibiotics before hospitalization were excluded.

### Microbiology

Methods for quantifying bacteria and *Candida* in sputum were described previously [10]. Briefly, sputum was solubilized with 0.4% N-acetyl cysteine, and ten-fold dilutions were made. Aliquots (0.01 ml) were streaked on blood and chocolate agar and incubated for 24–28 h at 37 °C in an atmosphere of 10% O_2_ and 5% CO_2_. WBC in solubilized sputum were quantified using a hemocytometer. The numbers of bacteria and *Candida* per ml sputum were calculated. Speciation was by conventional means and confirmed using MALDI-TOF.

### Case definitions

Our predetermined criterion for determining an etiologic role for a recognized bacterial pathogen, such as *Streptococcus pneumoniae, Haemophilus influenzae, Staphylococcus aureus*, or *Pseudomonas aeruginosa,* was the finding of > 10^5^ CFU/ml in a high-quality sputum sample [10, 12–15]. To designate an etiologic role for *Candida* spp.*,* we used more stringent criteria, as we have done for organisms that are usually reported as “normal respiratory flora” such as as viridans streptococci, *Corynebacteria*, or *Lactobacillus* [10, 16]: (1) Microscopic examination of Gram-stained sputum showed large numbers of yeast forms, including some within polymorphonuclear leukocytes; and (2) quantitative culture demonstrated > 10^6^ CFU *Candida* spp. per ml with or without other bacteria in numbers that met the above-stated criteria. In this observational study, laboratory studies, in addition to blood counts, usual blood chemistries, routine sputum and blood cultures, PCR on a nasopharyngeal swab for respiratory viruses, plasma procalcitonin, sputum WBC counts, and serum 1,3-beta-D glucan were done in a variable number of patients.

## Results

### Clinical findings

Six of 154 (3.9%) patients hospitalized for CAP had sputum samples that, based on the above microbiologic criteria, suggested a contributory etiologic role for *Candida* spp*.* The median age was 73 (range: 59 to 82). In every case the admitting history noted factors associated with aspiration, and aspiration pneumonia was included as a possible diagnosis (Table). Importantly, these cases were not selected because of a history suggesting aspiration but were identified by sputum criteria, after which the historical information at admission was determined from the medical record.

Patients identified in this fashion had a high rate of comorbid conditions: the average Charlson Comorbidity Index was 8 for patients with *Candida* pneumonia compared to 5.5 for patients with CAP due to other respiratory pathogens (*t*-test, *p* = 0.02) [10]*.* Two had well-controlled diabetes mellitus (hemoglobin A1c ≤ 6.7) and none was receiving glucocorticosteroids prior to admission. One of the patients was admitted directly from a hospice facility (case 5); the others were admitted from the community. None had intravenous lines at the time of admission, but 3 were receiving nutrition via percutaneous endoscopic gastrostomy tube. There was no documentation of antibiotics given in 8 weeks leading up to admission. In all cases, physical examination disclosed rales, and chest radiographs showed bilateral opacities. Thrush was noted in case 4 (Table). Computed tomography (CT) of the chest was performed in cases 4, 5, and 6. In all three, CT demonstrated bilateral patchy opacities consistent with multifocal pneumonia. Bronchiectasis was not noted. The peripheral WBC count at admission was elevated (> 10,500/mm^3^) in 4 cases. The median WBC count was 16,550/mm^3^, compared to median WBC counts of 12,800/mm^3^ in pneumococcal and 12,200/mm^3^ in *Haemophilus* pneumonia [17]. Plasma procalcitonin exceeded 0.5 ng/ml in 3 of 5 patients in whom it was tested.

### Microbiologic findings

**Fig. 1:**
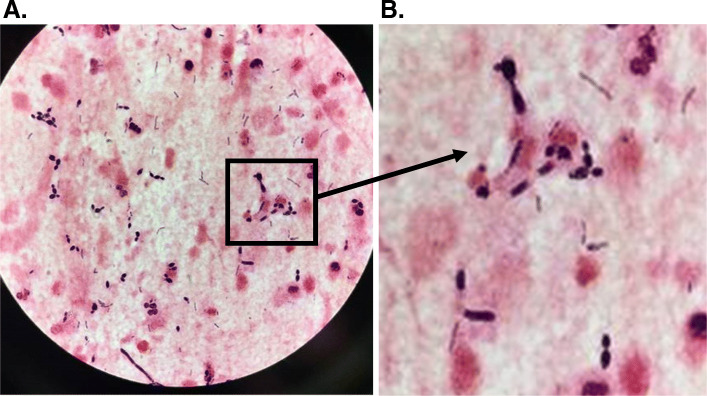
**A**. Sputum Gram stain (case 5) at 1000x magnification showing polymorphonuclear leukocytes and monocytes. The red background indicates protein in secretions. Absence of epithelial cells indicates absence of contamination by oropharyngeal secretions. Large numbers of budding yeast forms are seen, many of which are intracellular, with some pseudohyphae. Many fine Gram positive rods are also seen. **B**. Enlargement of boxed area shows what appear to be deteriorating pseudohyphae. Culture yielded 4X10^6^ *Candida tropicalis* and 1.6 × 10^8^ *Lactobacillus gasseri*

**Fig. 2:**
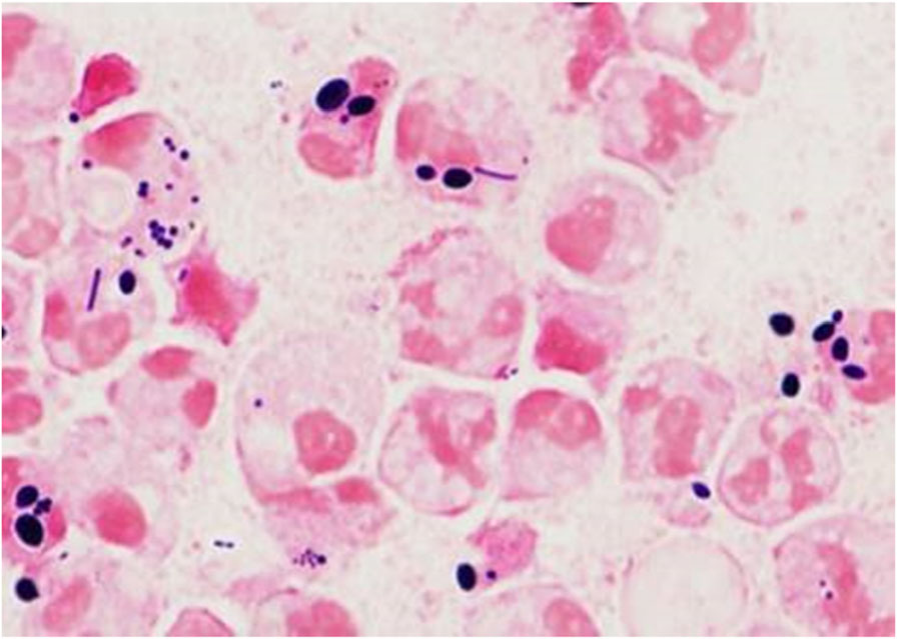
Sputum Gram stain (case 4) at 1000x magnification, showing many WBC with intracellular yeast and Gram positive cocci. Culture yielded 2 × 10^7^ *C. albicans,* 5 × 10^7^ *S. aureus*, and 4 × 10^7^ *S. pneumoniae*

In all 6 cases, large numbers of yeast forms were readily apparent on sputum Gram stain; 4–14% of polymorphonuclear cells (PMNs) contained intracellular yeast*,* and pseudohyphae were seen in 5 cases (Table, Figs. 1 and 2). These patients all had > 10^6^ CFU/ml of *Candida* spp. in their sputum: 3 with *C. albicans,* 2 with *C. glabrata,* and 2 with *C. tropicalis* (one specimen had both *C. tropicalis* and *C. glabrata*). However, no case met microbiologic criteria for pneumonia due to *Candida* alone: quantitative sputum cultures yielded > 10^5^ CFU/ml of a recognized bacterial pathogen (*S. pneumoniae, H. influenzae, S. aureus,* and/or *P. aeruginosa*) in 3 cases and > 10^6^ CFU/ml normal respiratory flora (*Lactobacillus*) in the other 3 cases. Gram stain and quantitative cultures yielded consistent results in all but 1 case in which many Gram positive cocci were seen but not cultured; we regarded these as probable anaerobic organisms [10]. The median number of WBC in sputum was 1.3 × 10^7^ per ml. An assay for serum 1,3-beta-D glucan was performed in 3 cases, and the level was > 500 ng/ml in all 3. Blood cultures, done in 6 patients, yielded *C. glabrata* in one case, but that patient also had *C. glabrata* in the urine and *C. albicans* in the sputum. PCR for respiratory viruses was positive in 2 of 3 cases in which it was done.

### Treatment

Anti-bacterial and anti-fungal treatments were given in all cases (fluconazole in 3 and micafungin in 3) (Table 1). Two patients ultimately had care withdrawn; the other 4 recovered. Mortality in these 6 patients was 33.3% during admission and 66.7% at 1 year.

**Table 1:** *Urine culture also grew *Candida glabrata*

Case	Sputum Gram Stain	Intracellular Yeast/Pseudohyphae	Sputum Culture CFU/ml	WBC/ml Sputum	Blood Culture	Viral PCR	ß-d Glucan pg/ml	Peripheral WBC mm^**3**^	PCT ng/ml	Chest Imaging	Aspiration Risk	Treatment	Outcome
1	Many WBC, many yeast, moderate GPR and GPC, rare GNR	Yes/Yes	*C. glabrata* 2 × 10^7^ *Pseudomonas* 2 × 10^5^	1.5 × 10^7^	*C. albicans*	ND	ND	17,800	17.7	Patchy bilateral opacities	Oropharyngeal dysphagia, PEG, previous admissions for aspiration	Piperacillin-tazobactam Micafungin	Opted for hospice and died within 1 month
2	Many WBC, yeast, GPR	Yes/Yes	*C. albicans* 5 × 10^6^ *Lactobacillus gasseri* 5 × 10^7^	1 × 10^7^	Negative	Influenza A	> 500	15,400	0.14	Diffuse bilateral opacities	Dysphagia, achalasia, documented aspiration	Vancomycin Ceftriaxone Metronidazole Micafungin	Died during admission
3	Many WBC, yeast, GPR, few GPC	Yes/No	C. albicans 6 × 10^6^ *Lactobacillus kalixensus* 4 × 10^6^	3.2 × 10^5^	Negative	ND	> 500	4200	< 0.09	Bilateral bibasilar opacities	Seizures, suspected aspiration	VancomycinCeftriaxone Metronidazole Trimethoprim-sulfamethaxole Fluconazole	Improved
4	Many WBC, yeast, GPC (pairs, chains, clusters)	Yes/Yes	*C. albicans* 2 × 10^7^ *S. aureus* 5 × 10^7^ *S. pneumoniae* 4 × 10^7^	9 × 10^7^	*S. aureus* C. glabrata*	Influenza A	> 500	17,700	ND	Diffuse bilateral opacities	PEG with pleasure feeds, suspected aspiration	Oseltamivir Piperacillin-tazobactam Nafcillin Fluconazole	Died during admission
5	Many WBC, yeast and GPR	Yes/Yes	*C. tropicalis* 4 × 10^6^ *Lactobacillus gasseri* 1.6 × 10^8^	ND	Negative	ND	ND	28,000	4.75	Bibasilar reticulonodular opacities	Tracheostomy, PEG, suspected aspiration	Vancomycin Cefepime Metronidazole Micafungin	Improved then died within 1 year
6	Many WBC, yeast, GPR	Yes/Yes	*C. glabrata* 1.3 × 10^8^ *C. tropicalis* 2 × 10^6^ *S. aureus* 5 × 10^5^	ND	ND	Negative	ND	4300	< 0.09	Bilateral opacities	Supraglottic squamous cell cancer, chronic dysphagia, suspected aspiration	CeftriaxoneAzithromycin Fluconazole	Improved

*Abbreviations*: *WBC* white blood cells, *PCT* procalcitonin, *CFU* colony forming units, *CCI* Charlson Comorbidity Index, *GPC* Gram positive cocci, *GPR* Gram positive rods, *GNR* Gram negative rods, *PMN* polymorphonuclear leukocytes, *ND* not done, *PEG* percutaneous endoscopic gastrostomy tube

## Discussion

In this prospective, observational study, we describe 6 of 154 patients in whom *Candida* spp. appeared to play a contributory etiologic role in CAP. These patients shared clinical and laboratory features that distinguished them from usual CAP patients. (1) All had conditions that suggested a diagnosis of aspiration pneumonia to their admitting physicians. (2) The Charlson Comorbidity Index was substantially higher than is usual for CAP patients. (3) Gram stains of sputum showed large numbers of yeast forms, many within PMNs; (4) Quantitative cultures yielded > 10^6^ CFU *Candida* per ml sputum. (5) Pseudohyphae were seen in 5 of 6 cases. (6) 1,3-beta-D-glucan was strongly positive in the 3 patients in whom it was tested.

Importantly, a *Candida* spp. was never detected as a sole infecting organism. Greater than 10^5^ CFU/ml of recognized bacterial pathogens or > 10^6^ CFU/ml of bacteria generally identified as ‘normal respiratory flora’ were also present in every case. Viral PCR was positive in 2 of 3 cases in which it was tested. In fact, 3 patients appeared to be infected with more than one Candida spp. In Case 6, two different Candida spp. were identified in sputum. In Cases 1 and 4, one Candida spp. was grown from blood and another was identified in the sputum. This seeming discrepancy results from the fact that only 1 or 2 colonies are selected for identification by MALDI-TOF. In young cultures, colonies of *C. albicans* and *C. glabrata* look alike, and the one growing in the blood might simply have been missed in the sputum. This explanation is supported by the finding of pseudohyphae in sputum from Case 1; the patient was infected with both, but only *C. glabrata*, which does not make pseudohyphae, was identified in sputum, whereas the blood culture yielded *C. albicans*. While these results do not prove that *Candida* spp. alone causes CAP, they suggest that *Candida spp.* may be a contributory cause of CAP, especially in patients who have a history of chronic aspiration.

Evidence opposing an etioloic role for *Candida* spp. in CAP has led to the teaching that the finding of *Candida* spp. in sputum culture simply reflects contamination by oropharyngeal colonization. *Candida* spp. regularly colonize the upper respiratory tract, with higher rates of colonization in sicker patients. An early study reported the presence of *Candida* in the sputum in increasing proportions of medical students, hospital employees, and patients, respectively [18]. Sputum cultures of 55% of medical inpatients yielded *Candida*; quantitative cultures were not done and the quality of the sputum sample was not addressed [18]. Rello et al reported that, in 28 adults with suspected pneumonia and positive sputum cultures for *Candida* spp., protected brush bronchoscopic specimens yielded > 10^3^ CFU/ml *Candida* spp. in 24 cases, but most of these were regarded as contaminants [19]. Of 135 autopsies done on patients with evidence of pneumonia, respiratory samples from 77 had been positive for *Candida* spp., but none had histologic evidence of *Candida* pneumonia [20]. Conversely, most patients with histologic evidence of *Candida* pneumonia on lung biopsy did not grow *Candida* on premortem cultures [21]. In contrast to our work, only one of these studies quantitated *Candida*, and used a cutoff of 10^3^ CFU/ml, lower than our cutoff of 10^6^ CFU/ml.

In patients with CAP, sputum is the expectorated material that has collected in alveoli, including plasma, PMNs, and microbes. A sample that shows pure, or nearly pure PMNs and large numbers of microbial forms should reflect alveolar exudate. We have used these criteria in the past to show that non-typeable *Haemophilus influenzae* [13], *Moraxella catarrhalis* [10, 22], *Corynebacterium* [16] and other bacteria generally dismissed by microbiology laboratories as ‘normal respiratory flora’ [10] all may cause pneumonia. To our knowledge, no one has previously reported Gram stains and quantitative cultures of *Candida* in high-quality sputum samples, while requiring large numbers of organisms including intracellular forms and pseudohyphae to be seen microscopically and > 10^6^ CFU/ml to be present. In the present study, the high quality of the sputum samples was shown by the presence of 1.3 × 10^7^ WBC per ml (median value) and the absence of epithelial cells in microscopic fields at 1000 x magnification. We identified intracellular yeast forms within PMNs in all samples and pseudohyphae in 5 samples, further supporting a pathogenic role for *Candida*.

Some of these approaches have been utilized in intubated patients with suspected ventilator-associated pneumonia (VAP). In one study, the presence of intracellular organisms in at least 2% of cells had a sensitivity of 84% and a specificity of 80% for VAP [23]. Using a cutoff of 5% intracellular organisms, Torres et al, found a positive predictive value of 75% for diagnosing VAP using protected bronchoalveolar lavage [24]. In another study, a cutoff of 7% was 97% specific for diagnosing VAP [25]. We observed intracellular yeast in every sputum sample.

A limitation of the current study is the absence of a diagnosis of *Candida* pneumonia by lung biopsy. Without histologic evidence of invasion of yeast into lung parenchyma, *Candida* pneumonia cannot be diagnosed with certainty. However, most etiologic diagnoses of pneumonia are presumptive, unless organisms are also grown from a normally sterile site. Additionally, not all patients had the same evaluation including 1,3-beta-D-glucan, procalcitonin, and viral PCR. Treatment decisions were made by managing clinicians; all patients received both antibacterial and antifungal agents, and care was withdrawn in 2 cases, making it impossible to determine which treatments were beneficial.

The present study provides evidence suggesting that, in patients who have risk factors for chronic aspiration *Candida* spp. plays a contributory role in the etiology of CAP. We found that patients with CAP who had large numbers of *Candida* in a high-quality sputum sample all had a history consistent with aspiration, and in each case the infection was polymicrobial. These findings support the principle that a sufficient inoculum of organisms of low virulence into the lower respiratory tract may suffice to cause pneumonia.

The subject of yeast-bacteria interaction has been extensively studied. Roux et al have shown in rats that infection with *Candida* facilitates bacterial infection by interfering with the function of alveolar macrophages [6, 8], and Neely et al [26] showed that bacterial colonization of burn wounds rendered *Candida* more invasive [7]. In critically ill patients, the presence of *Candida* in sputum cultures has been associated with increased risk of VAP due to *Pseudomonas aeruginosa* [27].

In conclusion, by examining sputum samples submitted for Gram stain and culture, we found that, of 154 high-quality specimens (> 20 WBC per epithelial cell) from patients who were hospitalized with a diagnosis of CAP, 6 contained large numbers of intra- and extracellular yeast forms. Admitting physicians, unaware of microscopic findings, diagnosed chronic aspiration in all cases. Quantitative cultures revealed > 10^6^ CFU *Candida* spp. per ml. Sputum from 5 samples showed pseudohyphae. One patient had candidemia, and in 3 whose serum was assayed for 1,3-beta-D-glucan, the level was markedly elevated. In every case bacterial coinfection was present and 2 patients had viral coinfection. These findings suggest that *Candida* spp. may play a contributory role in the etiology of CAP in patients who have a history of chronic aspiration. Future studies are needed to address whether patients with this constellation of findings benefit from antifungal therapy.

## Data Availability

The datasets used and/or analysed during the current study are available from the corresponding author on reasonable request.

## Abbreviations

CAP: Community-acquired pneumonia
WBC: White blood cells
CFU: Colony-forming units
PMN: Polymorphonuclear cells
VAP: Ventilator-associated pneumonia
